# Phenotyping of Human Pulp Cells After Cryopreservation

**DOI:** 10.1111/jcmm.71362

**Published:** 2026-09-16

**Authors:** Camila Maura Morais Lima dos Santos, Washington Henrique Themoteo da Silva, Fernanda Rodrigues Guedes, Milla Christian de Paula, Leslie Morse, Ricardo Battaglino, Marcelo José Barbosa Silva, Ana Paula Turrioni

**Affiliations:** ^1^ School of Dentistry Federal University of Uberlândia Uberlândia Minas Gerais Brazil; ^2^ Department of Orthopaedics University of Miami Miller School of Medicine Miami Florida USA; ^3^ Laboratory of Tumor Biomarkers and Osteoimmunology, Institute of Biomedical Sciences Federal University of Uberlândia Minas Gerais Brazil; ^4^ Department of Pediatric Dentistry and Orthodontics, School of Dentistry Federal University of Uberlândia Uberlândia Minas Gerais Brazil

**Keywords:** cryopreservation, dental pulp, differentiation, stem cells

## Abstract

This study aimed to compare cell viability, morphology, cell yield, and stem cell‐associated marker expression of cryopreserved human dental pulp cells (HDPCs) across different passages following thawing and serial in vitro expansion. HDPCs were isolated from three healthy donors, cryopreserved at −80°C for 3 months, and subsequently evaluated at different passages. Non‐cryopreserved passage 0 (P0) cells were used as the baseline control. Cell viability was assessed using the MTT assay, cell morphology and complementary cell counting by scanning electron microscopy (SEM), and phenotypic marker expression (CD44, CD146, STRO‐1, and CD45) by flow cytometry. P6 and P10 showed significantly higher cell viability than P0 and P12 (*p* < 0.05), whereas SEM‐based cell counting showed a higher number of cells at P6 than at P0, P3, and P12 (*p* < 0.05). The percentage of CD146‐positive cells decreased from 32.1% and 32.3% at P0 and P3, respectively, to 4.9% and 5.2% at P10 and P12 (*p* < 0.05). A similar passage‐dependent reduction was observed for STRO‐1 expression. Overall, HDPCs exhibited passage‐dependent changes following cryopreservation, thawing, and serial expansion. Intermediate passages, particularly P6, showed favourable cell viability and yield, whereas earlier passages better preserved progenitor‐associated phenotypic markers. These findings highlight the importance of passage selection when cryopreserved HDPCs are expanded for subsequent experimental applications.

## 1 Introduction

The dental pulp is a highly vascularized and innervated soft connective tissue located within the pulp chamber and root canals of the tooth. It comprises odontoblasts, fibroblasts, immune cells, and a resident population of multipotent stem/progenitor cells [1]. Dental pulp stem cells (DPSCs) were first isolated and characterized in 2000 as a highly clonogenic and rapidly proliferating cell population capable of generating dentine–pulp‐like complexes following in vivo transplantation [2]. Since then, DPSCs have been recognized as a distinct population of mesenchymal stem cells that shares key biological characteristics with bone marrow‐derived mesenchymal stem cells while offering the advantage of an easily accessible and minimally invasive cell source, typically obtained from extracted third molars or exfoliated deciduous teeth [3]. Owing to their high proliferative capacity, multilineage differentiation potential, and immunomodulatory properties, DPSCs have emerged as promising candidates for applications extending beyond dental and craniofacial regeneration, including bone, cartilage, neural, and vascular tissue engineering [4]. Therefore, preserving the biological characteristics of human dental pulp cells (HDPCs) during in vitro expansion and cryopreservation is essential to maintain their regenerative capacity and therapeutic potential.

The biological behaviour of HDPCs, however, is strongly influenced by in vitro expansion conditions, particularly the number of cell passages [5]. Previous studies have demonstrated that prolonged passaging may alter proliferation rates, senescence profiles, and phenotypic marker expression, potentially compromising cellular function and therapeutic applicability [6, 7, 8]. Despite this, there is still no consensus regarding the most appropriate passage stage for experimental or clinical use of dental pulp–derived cells.

In parallel, cryopreservation plays a critical role in cell banking, experimental reproducibility, and future clinical translation [9, 10, 11]. Cryopreservation protocols for dental pulp stem cells differ with respect to freezing procedures, cryoprotectant formulations, and storage conditions. Although storage in liquid nitrogen remains the gold standard for long‐term preservation of biological specimens, several studies have demonstrated that dental pulp‐derived cells can also be successfully preserved at −80°C [8]. In particular, cryopreservation using 10% dimethyl sulfoxide (DMSO) as a cryoprotectant combined with storage at −80°C has been shown to maintain favourable cell viability and key biological characteristics of human dental pulp stem cells after 6 and 12 months of storage [12]. Likewise, simplified cryopreservation protocols have yielded satisfactory recovery of dental pulp stem cells, supporting the feasibility of accessible and cost‐effective strategies for dental stem cell banking [13]. Nevertheless, the effects of −80°C cryopreservation on the biological characteristics of human dental pulp cells at different stages of in vitro expansion remain poorly understood.

Although prior investigations suggest that dental pulp–derived cells may retain viability and phenotypic traits following cryostorage [14, 15], most studies have evaluated isolated passages or have not systematically examined the combined effects of cryopreservation and passage number on HDPC phenotype. For instance, cryopreserved HDPCs at the third passage have been reported to exhibit significant expression of mesenchymal stem cell–associated markers, including STRO‐1, CD105, and CD90, alongside negative expression of the haematopoietic marker CD45 [16]; however, it remains unclear how these markers behave across subsequent passages following cryopreservation and in vitro expansion. Comprehensive characterization of HDPCs across multiple passage stages following cryopreservation therefore remains limited, leaving an important gap in defining how cryostorage influences cellular quality, stemness‐related marker expression, and expansion‐related changes over time.

Thus, there is still a need for studies that provide a more detailed, passage‐by‐passage characterization of HDPCs following cryopreservation, in order to support their standardized application in tissue repair and regeneration. In this context, the objective of the present study was to compare cell viability, proliferative behaviour, morphology, and stem cell markers of cryopreserved human dental pulp cells (HDPCs) across different passages following thawing and in vitro expansion.

## 2 Methodology

**FIGURE 1:**
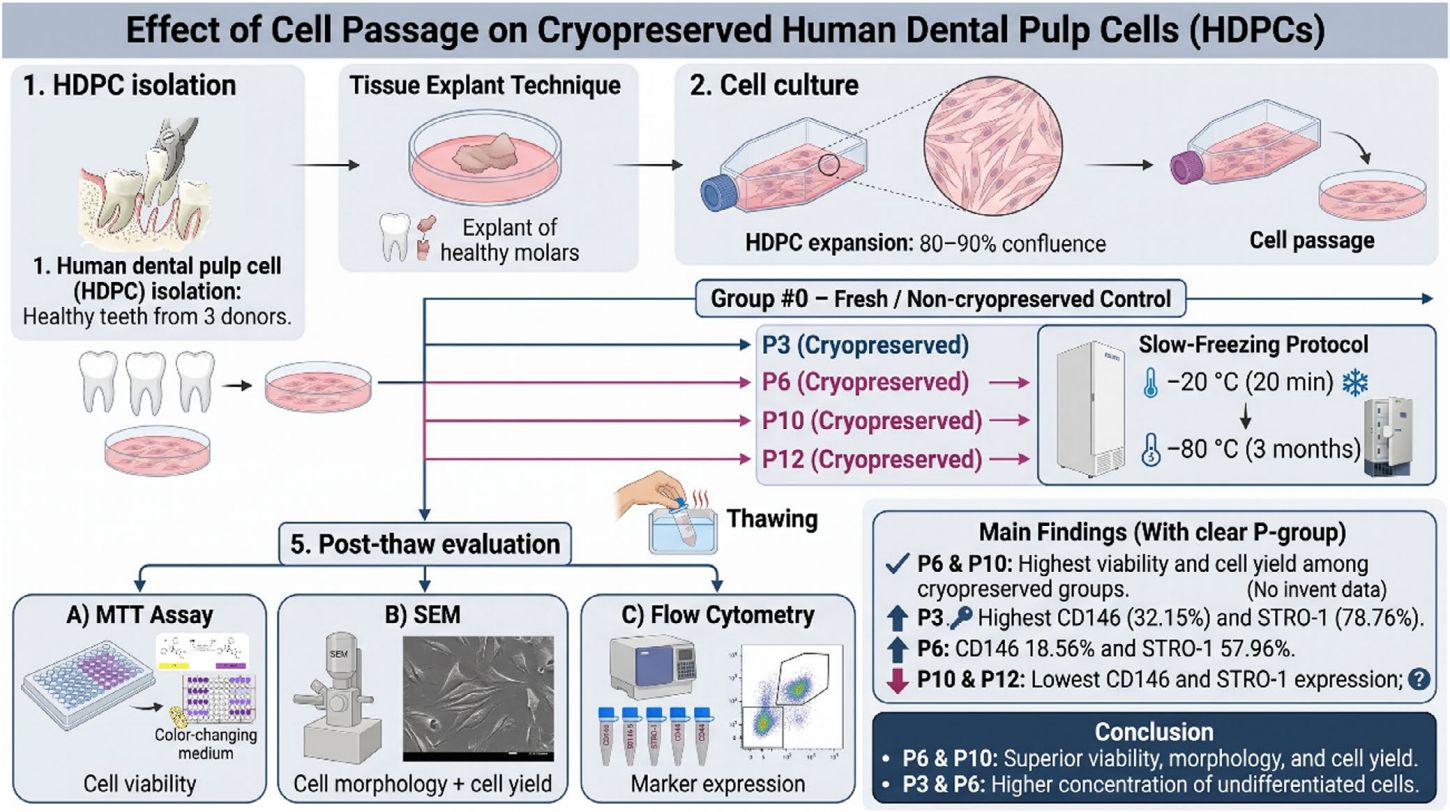
Schematic representation of the experimental workflow. Human dental pulp‐derived cells (HDPCs) were isolated and cryopreserved using a freezing medium containing 10% dimethyl sulfoxide (DMSO). After thawing, the cells were expanded in vitro and evaluated at different passages. Cell viability, morphology, proliferation, and immunophenotypic characteristics were subsequently assessed to determine the effects of cryopreservation during in vitro expansion.

A schematic overview of the experimental workflow, including cell isolation, cryopreservation, thawing, cell culture, and the subsequent experimental analyses, is presented in Figure 1.

### 2.1 Obtaining Human Dental Pulp Cells (HDPCs)

This study was conducted in accordance with the Declaration of Helsinki and was approved by the Research Ethics Committee involving Human Beings at the Federal University of Uberlândia (CAAE: 18148819.9.0000.5152), following the regulations of the Brazilian National Health Council. Patients were informed about the research, risks, and benefits, and signed the Informed Consent Form (ICF) before the procedure. Human dental pulp cells (HDPCs) were isolated from three third molars, each obtained from a different donor, in order to account for inter‐individual genetic variability, as recommended in previous reports [17]. HDPCs were obtained from three independent teeth from three different donors (*n* = 3 biological samples) [18]. The number of wells and technical measurements varied according to each experimental assay.

The dental pulp was carefully washed with a phosphate‐buffered saline solution (PBS, Invitrogen, Carlsbad, CA, USA), quickly dissected, and placed in a 35 mm culture Petri dish (Corning Inc., Corning, NY, USA). The tissue explant was cultured in DMEM/F‐12 (DMEM/F‐12, Invitrogen Corporation—Carlsbad, CA, USA) supplemented with 15% FBS, 100 U/mL penicillin, 100 U/mL streptomycin, 2 mM L‐glutamine, and 2 mM non‐essential amino acids (Invitrogen Corporation—Carlsbad, CA, USA) in a controlled environment containing 5% CO_2_ at 37°C. After 3–4 days, fibroblast‐like cells began to form from the tissue explants adhered to the plate. Upon identification of these cells, the explant was transferred to another Petri dish under the same culture conditions. This procedure was repeated several times. The cell monolayers were washed twice with PBS and exposed to 0.5 g/L trypsin and 0.53 mmol/L ethylenediaminetetraacetic acid (EDTA) (Invitrogen Corporation—Carlsbad, CA, USA) for 3 to 5 min at 37°C. Trypsin inactivation was performed by adding culture medium with 10% FBS. After this step, the cells were transferred to a 25 cm^2^ flask (Corning Inc., Corning, NY, USA).

### 2.2 Cell Cryopreservation Protocol

For passages 3,6, 10 and 12 HDPCs at 80%–90% confluence, were detached using 0.25% trypsin–EDTA (T4049, Sigma‐Aldrich, St. Louis, MO, USA) for 3–6 min, depending on the culture flask size. Enzymatic activity was neutralized with pre‐warmed complete Dulbecco's Modified Eagle Medium (DMEM; Gibco, Thermo Fisher Scientific, Waltham, MA, USA) supplemented with 10% fetal bovine serum (FBS). Cell suspensions were centrifuged at 1200 rpm for 2 min using a bench‐top centrifuge (Daiki, fixed‐angle rotor for 12 × 15 mL conical tubes), and the resulting cell pellet was gently resuspended in freezing medium composed of 80% DMEM, 10% FBS, and 10% dimethyl sulfoxide (DMSO) at a final concentration of 1–2 × 10^6^ cells/mL.

Aliquots of 1 mL of the cell suspension were transferred into sterile cryovials. Cryopreservation was performed using a stepwise slow‐freezing protocol adapted from previous studies [14, 19]. Cryovials were initially placed in a −20°C vertical freezer (Brastemp Frost Free Vertical Freezer Flex Eletrônico, 280 L, Brazil) for 20 min to promote gradual cooling, followed by transfer to an ultra‐low temperature freezer (CL120‐86 V, Coldlab, Brazil) maintained at −80°C, where samples were stored for 3 months. This stepwise cooling procedure was adopted to minimize thermal and osmotic stress during freezing, thereby reducing intracellular ice crystal formation and improving post‐thaw cell recovery.

Cells at passage 0 (P0), prior to cryopreservation, were used as the non‐cryopreserved control group and served as the reference condition for comparison with cryopreserved cells evaluated at subsequent passages. Thus, P0 cells represented the baseline cellular condition before exposure to cryopreservation and subsequent in vitro expansion procedures.

### 2.3 Cell Thawing

Cryovials were rapidly thawed in a 37°C water bath until completely thawed. The cell suspension was gently homogenized and immediately transferred into a 25 cm^2^ culture flask containing 5 mL of pre‐warmed complete DMEM supplemented with 20% fetal bovine serum (FBS). Cells were cultured in a humidified CO_2_ incubator (Forma Series 3 Water Jacket CO_2_ Incubator, Thermo Scientific, Waltham, MA, USA) at 37°C with 5% CO_2_ and 95% humidity.

### 2.4 MTT Assay

MTT assay was conducted at 5 time points: initially at passage 0 and subsequently at passages 3, 6, 10, and 12 after 3 months of cryopreservation at −80°C. Cells from the three independent biological samples were seeded in 96‐well plates at a density of 10,000 cells per well, using nine wells per experimental condition (*n* = 9 wells). Cells were maintained for 24 h in a humidified CO_2_ incubator at 37°C and subsequently subjected to the MTT assay. Briefly, cells were incubated with MTT solution (3‐[4,5‐dimethylthiazol‐2‐yl]‐2,5‐diphenyltetrazolium bromide) at 37°C for 4 h. The resulting formazan crystals were then dissolved in DMSO, and absorbance was measured at 570 nm using the GLOMAX multimodal microplate reader (Promega Corporation, São Paulo, Brazil). Cell viability was determined based on the optical density values obtained [17].

### 2.5 Flow Cytometry

**FIGURE 2:**
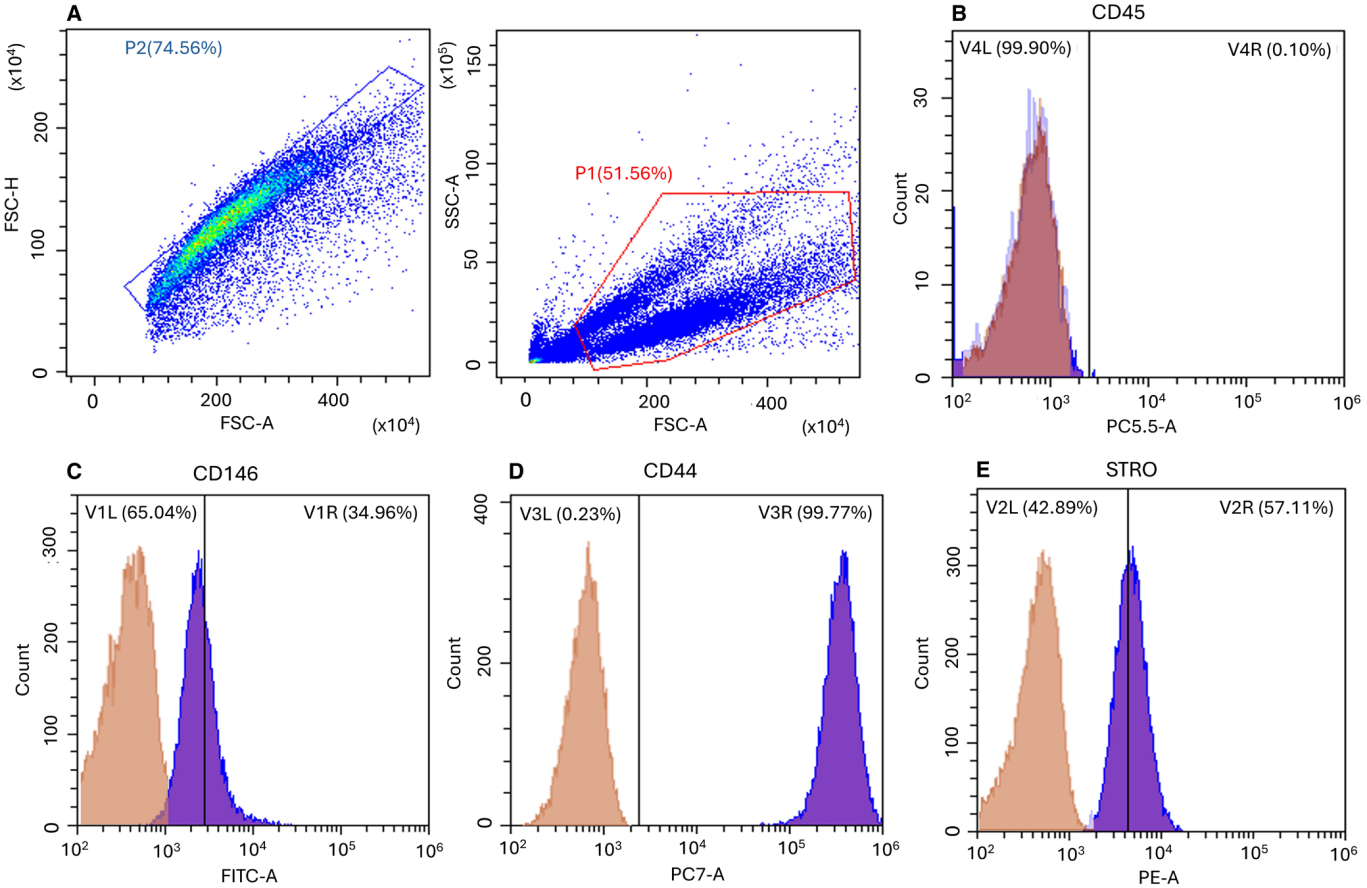
(a) Flow cytometry gating strategy used to exclude cellular debris and doublets. (b) Negative control marker (CD45). Representative expression profiles of the stem cell‐associated markers (c) CD44, (d) CD146, and (e) STRO‐1. Data are shown for cells at passage 3 from one representative patient.

Flow cytometry was conducted at 5 time points: initially at passage 0 and subsequently at passages 3, 6, 10, and 12 after 3 months of cryopreservation at −80°C. Cells (100,000) were seeded in 25 cm^2^ Petri dishes (*n* = 6 wells). Cells were maintained for 24 h in a humidified CO_2_ incubator at 37°C and subsequently subjected to the protocol. The staining panel comprised 4 fluorochrome‐conjugated antibodies (CD44, CD146, STRO‐1, and CD45‐ Abcam inc, Waltham, MA, USA). Gating was performed using FSC‐A/FSC‐H plots to exclude doublets and aggregates and SSC‐A/FSC‐A plots to select specific populations. Negative control (CD45) and stem cell markers (CD44, CD146, STRO‐1) were analysed (Figure 2).

Cells were treated with trypsin, centrifuged, and washed with 0.1% BSA in PBS at 4°C, followed by fixation with 4% paraformaldehyde and permeabilization using 0.1% Triton X‐100. Cells were incubated with primary antibodies (50 μL) for 1 h at room temperature, washed, and incubated with secondary antibodies for 45 min in the dark. After final washes, samples were analysed using a FACS flow cytometer (Becton, Dickinson) with Quest CELL software (Becton, Dickinson).

### 2.6 Morphological Analysis—Scanning Electron Microscopy (SEM)

Qualitative and quantitative morphological analysis. After 3 months of cryopreservation, cells from different passages were thawed and seeded onto glass coverslips in 24‐well plates at a density of 50,000 cells per well (*n* = 2 wells). After 24 h of incubation, samples were fixed in 2.5% glutaraldehyde (Sigma‐Aldrich) for 1 h and subsequently dehydrated through a graded ethanol series. The specimens were then mounted on aluminium stubs and sputter‐coated with a thin gold layer to enhance surface conductivity. Morphological evaluation was performed using a scanning electron microscope (SEM; Tescan Vega 3 LMU, Oxford Instruments, Abingdon, UK) operated at an accelerating voltage of 15 kV under high‐vacuum conditions. Images were acquired using a secondary electron (SE) detector, which is suitable for assessing surface morphology of fibroblast‐like cells. For each specimen, micrographs were obtained at 200× magnifications from four representative regions (*n* = 6). Digital images were captured and analysed using ImageJ software for quantitative cell counting. The analysis was conducted by three calibrated, single‐blind examiners. Intra‐examiner reliability ranged from *κ* = 0.89 to 0.91, and inter‐examiner reliability ranged from *κ* = 0.85 to 0.89. SEM analysis was primarily performed to qualitatively assess cell morphology and attachment at the different passages. In addition, the same SEM images were used to obtain complementary quantitative data on cell number. The same sampling criteria and magnification were applied to all experimental groups to ensure consistency across measurements. SEM‐based cell counting was considered a complementary quantitative assessment. All SEM micrographs were acquired under standardized imaging conditions and included scale bars for dimensional reference.

### 2.7 Statistical Analysis

Technical replicates were summarized for each donor and experimental condition, and the resulting donor‐level values were used for statistical analysis. The donor‐derived cell culture was considered the biological experimental unit (*n* = 3). Since the same donor‐derived cultures were evaluated across different passages and the data did not meet the assumptions of normality, differences among passages were analysed using the Friedman test for repeated measures. When a significant overall effect was detected, pairwise comparisons between passages were performed using the Wilcoxon signed‐rank test with Holm adjustment for multiple comparisons. Statistical significance was set at *p* < 0.05. All analyses were performed using SPSS software 25.0.

## 3 Results

### 3.1 Cell Passages 6 and 10 Presented the Best Results for Viability and Quantity of Pulp Cells

**FIGURE 3:**
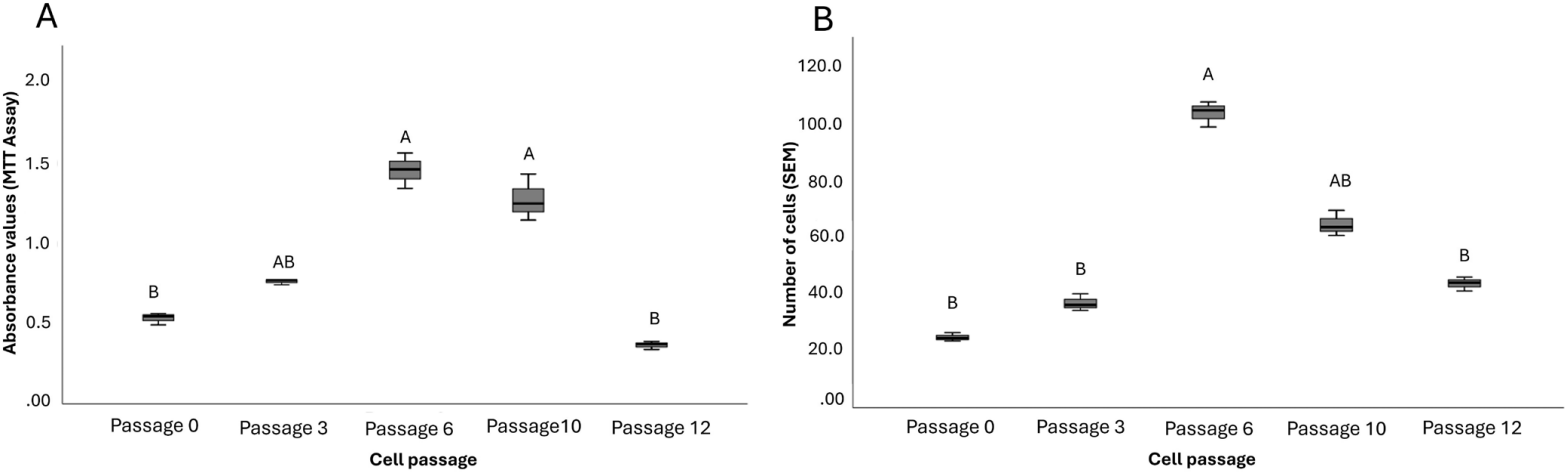
Box‐plots displaying the absolute absorbance values data from the cell viability tests (A) and SEM cell counts (B) of human dental pulp cells for passages 3, 6, 10, and 12 after 3 months of cryopreservation at −80°C. GLM Repeated Measures, *p* < 0.05 (*n* = 3). Different letters indicate statistically significant differences between groups.

Figure 3 presents the quantitative results from cell viability (MTT Assay) and number of cells detected by SEM. Cell viability (Figure 3a) differed significantly among passages, with P6 and P10 showing the highest viability values. Cell viability at P6 was significantly higher than that observed at P0 (*p* = 0.019) and P12 (*p* = 0.017). Similarly, P10 showed significantly higher cell viability compared with P0 (*p* = 0.022) and P12 (*p* = 0.019).

**FIGURE 4:**
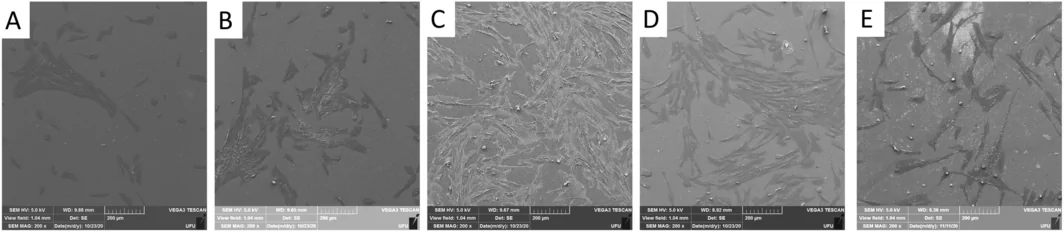
Cell morphology (SEM) in passages 0 (A), 3 (B), 6 (C), 10 (D), and 12 (E), at 200× magnification. SEM HV 5.0 kV, view field 1.04 mm.

Regarding the number of cells observed by SEM (Figure 3b), P6 showed a significantly higher cell count compared with P0 (*p* = 0.015), P3 (*p* = 0.017), and P12 (*p* = 0.026). Additionally, the cell morphology evaluation identified fibroblast‐like pulp cell shapes with odontoblastic extensions adhered to the glass coverslip substrate in all passages. However, a greater number of cells was observed in passages 6 and 10 (Figure 4).

### 3.2 Cell Passages 0 and 3 Presented the Highest Concentration of Stem Cells Markers

**FIGURE 5:**
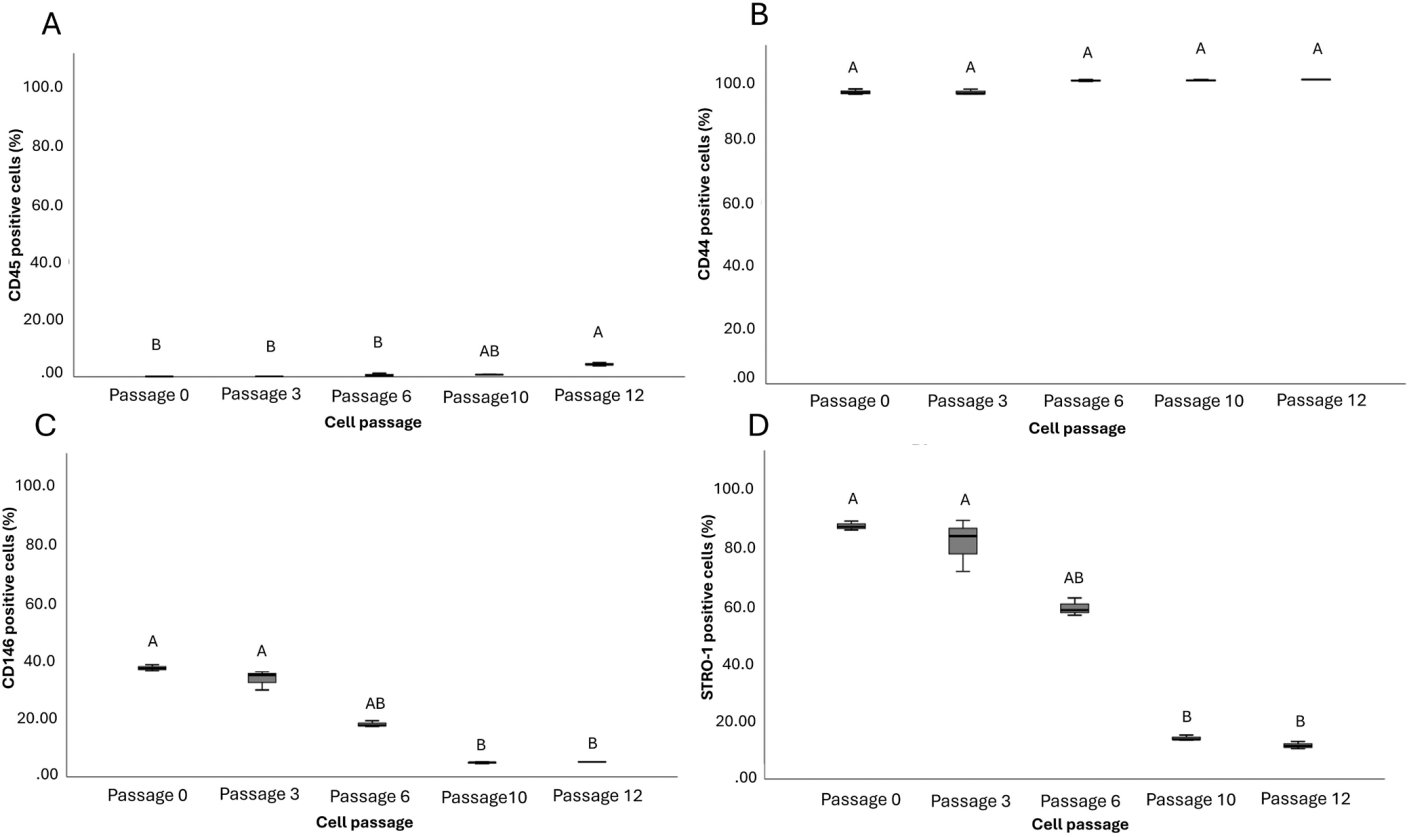
Box‐plot graph and table indicating the results of antibody markers CD45 (A), CD44 (B), CD146 (C), and STRO‐1 (D) (*n* = 3). GLM Repeated Measures, *p* < 0.05. Different letters indicate statistically significant differences between groups.

Flow cytometry results are presented in Figure 5, showing each antigen marker and the corresponding cell passages. Human dental pulp cells were used for the non‐storage group (0) and for different passages after 3 months of cryopreservation at −80°C.

For CD45 positive cells (Figure 5a), there was no significant difference between passages 3, 6, and 10, with medians of 0.1%, 0.5%, and 0.8% for CD45. Passage 12 differed from passage 0 (*p* = 0.019), passage 3 (*p* = 0.0017), and passage 6 (*p* = 0.022) with a median labeling of 4.85%. For CD44 positive cells (Figure 5b), there was no statistical difference among groups (*p* > 0.05).

For CD146 expression (Figure 5c), the percentage of CD146‐positive cells was highest at P0 and P3, with median values of 32.1% and 32.3%, respectively. CD146 expression decreased at later passages, with median values of 4.9% at P10 and 5.2% at P12. The percentage of CD146‐positive cells was significantly lower at P10 and P12 compared with P0 and P3 (P0 vs. P10, *p* = 0.019; P0 vs. P12, *p* = 0.013; P3 vs. P10, *p* = 0.022; P3 vs. P12, *p* = 0.024). No significant differences were observed among the remaining pairwise comparisons.

The percentage of STRO‐1‐positive cells (Figure 5d) was significantly higher at P0 and P3 than at P10 and P12. Significant differences were observed between P0 and P10 (*p* = 0.017), P0 and P12 (*p* = 0.017), P3 and P10 (*p* = 0.023), and P3 and P12 (*p* = 0.019). No significant differences were observed among the remaining pairwise comparisons.

## 4 Discussion

It is important to define the ideal conditions considering the isolation and applications of dental pulp stem cells (DPSCs) in different therapies, as factors such as donor age, passage number, and techniques can interfere with the quality and phenotype of the cells. In the present study, passage number during cryopreservation at −80°C for 3 months significantly impacted cell viability, yield, and the expression of CD146, STRO‐1, and CD45. Although SEM analysis confirmed preservation of fibroblast‐like morphology across groups, progressive immunophenotypic shifts were detected, including reduced CD146 and STRO‐1 expression and a relative increase in CD45 in later passages. These findings indicate that molecular alterations may precede detectable structural changes, reinforcing that morphological assessment alone is insufficient to define biological stability. This increase in CD45 expression, although still limited, may reflect stress‐induced phenotypic modulation or the emergence of minor non‐mesenchymal subpopulations during extended culture and post‐thaw recovery, rather than a complete loss of mesenchymal identity [20].

According to the International Society for Cellular Therapy (ISCT), DPSCs exhibit the characteristic mesenchymal stem cell (MSC) immunophenotype, including expression of CD73, CD90, and CD105 and absence of haematopoietic markers such as CD45 [21]. Previous studies have demonstrated that DPSCs can maintain high viability, proliferative capacity, mesenchymal immunophenotype, and osteogenic and adipogenic differentiation potential following long‐term cryopreservation [9, 10], suggesting that cryostorage does not necessarily compromise their fundamental stem cell properties. Consistent with these findings, DPSCs cryopreserved with 10% DMSO and stored at −80°C for 6–12 months have shown preservation of stemness‐related markers and proliferative capacity comparable to fresh controls [11]. Moreover, simplified protocols that do not require controlled‐rate freezing or specialized equipment have enabled successful DPSC recovery while preserving relevant biological characteristics [13]. Studies using different uncontrolled‐rate freezing approaches have similarly reported maintenance of key DPSC properties after cryopreservation, although these evaluations were generally restricted to a single, predefined cell passage [19]. In contrast, the present study evaluated cryopreserved cells across serial passages after thawing, allowing the identification of passage‐dependent phenotypic changes. Although the overall mesenchymal phenotype was maintained, CD146 and STRO‐1 expression progressively decreased at later passages, with significantly lower levels at P10 and P12 compared with earlier passages. These findings suggest that progenitor‐associated phenotypic characteristics may be more sensitive to prolonged post‐thaw expansion than the broader mesenchymal phenotype. However, because passage‐matched non‐cryopreserved controls were not available, the relative contributions of cryopreservation and serial passaging to these changes cannot be fully distinguished.

This trend is consistent with previous reports showing a progressive decline in CD146 expression with increasing passage number in dental pulp‐derived mesenchymal stem cells. CD146 expression has been reported to decrease gradually from passage 5 to passage 20, with a statistically significant reduction at passage 20 compared with passage 5. Notably, CD146 has been proposed as a functional marker of stem cell potency because of its association with proliferative, differentiation, and immunoregulatory capacities [22]. These findings are consistent with our observation of reduced CD146 and STRO‐1 expression at later passages (P10 and P12) compared with earlier passages (P3 and P6), suggesting a progressive loss of stemness‐associated characteristics during prolonged in vitro expansion. More broadly, extended cell culture has been associated with replicative senescence, increased apoptosis, and progressive phenotypic alterations [10]. Importantly, functional deterioration may occur at advanced passages even when conventional MSC‐associated markers remain detectable [23], highlighting that maintenance of the basic immunophenotypic profile does not necessarily indicate preservation of the cells' original biological properties. In our study, this reduction suggests progressive depletion of progenitor‐enriched subpopulations during serial expansion and subsequent freezing. Given that CD146 is associated with perivascular stem cell niches and clonogenic potential, and STRO‐1 represents an early mesenchymal progenitor marker, their decline may reflect selective expansion of more differentiated fibroblast‐like populations rather than complete loss of mesenchymal identity [22, 24].

The absence of marked ultrastructural differences under SEM contrasts with the immunophenotypic shifts observed, supporting the concept that molecular instability can emerge prior to visible morphological alterations during stem cell aging and manipulation [9]. In the present study, early passage cells (passage 3) maintained comparable behaviour before and after cryopreservation (*p* > 0.05), suggesting greater biological resilience at earlier expansion stages. Nevertheless, the combined effect of serial passaging and rapid freezing protocols may potentiate sublethal cellular stress.

Our finding that cell viability and yield were highest at intermediate passages (P6 and P10) broadly aligns with previous evidence indicating that DPSC proliferative performance is better preserved at earlier and intermediate passages and tends to decline with prolonged in vitro expansion. A previous study comparing early (P5), extended (P10), and late (P15) passages reported a marked passage‐dependent reduction in proliferation, with P15 cells exhibiting approximately 50%–70% lower growth than P5 cells [11]. Similarly, DPSCs have been reported to maintain high viability and functional integrity up to passage 14, whereas membrane damage and pre‐apoptotic changes became more evident at later passages, supporting the preferential use of earlier passages for experimental and potential therapeutic applications [25]. Regarding cell morphology, a progressive transition from the typical spindle‐shaped phenotype to enlarged, polygonal, and increasingly heterogeneous cells has also been described with advancing passage number [11]. Although these morphological changes were assessed by optical microscopy rather than SEM, they are consistent with our qualitative observations at higher passages. Taken together, these findings suggest that progressive alterations in proliferative behaviour and cellular morphology are characteristic of prolonged in vitro expansion of DPSCs and may occur irrespective of cryopreservation history or the imaging modality used for morphological assessment.

Cryopreservation methodology also contributes to variability. Although dimethyl sulfoxide (DMSO) remains widely used to limit intracellular ice formation, rapid freezing can induce osmotic imbalance and stress responses that may influence cellular phenotype [26]. Thus, phenotypic alterations observed in later passages likely reflect the interaction between replicative history and cryostorage‐related stress rather than freezing alone. In addition, storage at −80°C, as performed in the present study, is a widely used and accessible approach for short‐ to medium‐term cryopreservation and has been shown to preserve overall cell viability and mesenchymal characteristics. These findings support the idea that, although basic cellular properties are preserved, more sensitive progenitor features may be progressively affected under these conditions. Findings indicate that while DPSCs preserve fundamental mesenchymal characteristics following short‐term cryostorage, passage number critically modulates phenotypic stability. Intermediate passages appear to better maintain biological attributes after freezing. Nonetheless, extended storage periods, functional differentiation assays, and molecular profiling are required to confirm long‐term stability and translational applicability.

The absence of passage‐matched non‐cryopreserved controls represents a limitation of this study, as it prevents the effects of cryopreservation from being fully distinguished from those of serial cell expansion. However, the present study primarily aimed to compare the biological characteristics of cryopreserved dental pulp‐derived cells across different passages after thawing and serial expansion. Aditionally, SEM‐based cell counting should be interpreted as a complementary quantitative assessment rather than a direct measure of cell proliferation, and future studies may incorporate additional proliferation‐specific assays to further characterize passage‐dependent cellular behaviour.

## 5 Conclusion

Intermediate passages of cryopreserved human dental pulp cells demonstrated more favourable biological performance. Passages 6 and 10 showed higher viability, cell yield, and preserved fibroblast‐like morphology after 3 months of storage at −80°C, while passages 3 and 6 exhibited greater expression of stemness‐associated markers (CD44, CD146, and STRO‐1). These results indicate that passage number influences the quality and phenotypic stability of cryopreserved HDPCs. However, further studies with extended storage periods, additional functional assays, and larger sample sizes are needed to confirm these findings and support translational applications.

## Author Contributions

**Camila Maura Morais Lima dos Santos:** conceptualization, investigation, methodology, data curation, writing – original draft, writing – review and editing. **Washington Henrique Themoteo da Silva:** data curation, methodology, visualization, writing – review and editing. **Fernanda Rodrigues Guedes:** investigation, methodology, writing – review and editing. **Milla Christian de Paula:** investigation, methodology, validation, writing – review and editing. **Leslie Morse:** conceptualization, resources, supervision, visualization, writing – review and editing. **Ricardo Battaglino:** resources, supervision, visualization, writing – review and editing. **Marcelo José Barbosa Silva:** conceptualization, supervision, visualization, methodology, resources, writing – review and editing. **Ana Paula Turrioni:** conceptualization, supervision, project administration, funding acquisition, resources, writing – review and editing.

## Funding

Coordenação de Aperfeiçoamento de Pessoal de Nível Superior—Brasil (CAPES)—Finance Code 001, Conselho Nacional de Desenvolvimento Científico e Tecnológico (CNPq—434204/2018‐8, 307974/2019‐7, INCT 406840/2022‐9), and Fundação de Amparo a Pesquisa do Estado de minas Gerais (FAPEMIG—APQ00927‐23 and RED‐00204‐23).

## Ethics Statement

This study was conducted in accordance with the Declaration of Helsinki and was approved by the Research Ethics Committee involving Human Beings at the Federal University of Uberlândia (CAAE: 18148819.9.0000.5152), following the regulations of the Brazilian National Health Council.

## Consent

All patients provided their written informed consent.

## Conflicts of Interest

The authors declare no conflicts of interest.

## Data Availability

The data that support the findings of this study are available from the corresponding author upon reasonable request.
